# Quercetin Attenuates d-GaLN-Induced L02 Cell Damage by Suppressing Oxidative Stress and Mitochondrial Apoptosis *via* Inhibition of HMGB1

**DOI:** 10.3389/fphar.2020.00608

**Published:** 2020-05-05

**Authors:** Peng Fang, Jiajun Liang, Xuejiao Jiang, Xian Fang, Mengli Wu, Xiaoyi Wei, Wenlong Yang, Weixin Hou, Qiuyun Zhang

**Affiliations:** Beijing Key Laboratory of TCM Collateral Disease Theory Research, School of Traditional Chinese Medicine, Capital Medical University, Beijing, China

**Keywords:** quercetin, high mobility group box-1, d-galactosamine, oxidative stress, apoptosis

## Abstract

High mobility group box-1 (HMGB1) plays an important role in various liver injuries. In the case of acute liver injury, it leads to aseptic inflammation and other reactions, and also regulates specific cell death responses in chronic liver injury. HMGB1 has been demonstrated to be a good therapeutic target for treating liver failure. Quercetin (Que), as an antioxidant, is a potential phytochemical with hepatocyte protection and is also considered to be an inhibitor of HMGB1. However, the mechanism of its hepatoprotective effects remains to be characterized. The present study explored whether the hepatoprotective effect of Que antagonizes HMGB1, and subsequent molecular signaling events. Our results indicated that Que protects L02 cells from d-galactosamine (d-GaLN)-induced cellular damage by reducing intracellular reactive oxygen species (ROS) production and apoptotic responses in the mitochondrial pathway. Immunofluorescence and Western blot assays showed that HMGB1 was involved in d-GaLN-induced L02 cell damage. Further research showed that after transfection with HMGB1 short hairpin RNA (shRNA), cell viability was improved, and intracellular ROS production and apoptosis were suppressed. When co-treated with Que, the expression of HMGB1 was decreased significantly, the expression of proteins in the corresponding signal pathway were further reduced, and the production of ROS and apoptosis were further suppressed. Molecular docking also indicated the binding of Que and HMGB1. Taken together, these results indicate that Que significantly improves d-GaLN-induced cellular damage by inhibiting oxidative stress and mitochondrial apoptosis *via* inhibiting HMGB1.

## Introduction

High mobility group box-1 (HMGB1) is an evolutionarily conserved nuclear DNA-binding protein widely found in eukaryotic cells, which has multiple conflicting functions (both inflammatory and cell protective), depending on its location (Kang et al., 2014; Musumeci et al., 2014). HMGB1 acts as a damage-associated molecular pattern (DAMP) molecule, when it is passively released after cell damage or actively secreted into the extracellular space, it communicates the occurrence of injury and inflammation to neighboring cells *via* the receptor for advanced glycation end products (RAGE) or toll-like receptor 4 (TLR-4) (Scaffidi et al., 2002; Huebener et al., 2015). HMGB1 contributes to aseptic inflammation and other responses in acute liver injury, playing a key role (Yang et al., 2017). It is also an important hepatocyte DAMP, which regulates specific cell death responses in chronic liver injury (Hernandez et al., 2018). Studies have shown that serum HMGB1 levels in patients with acute or chronic liver failure (ACLF) are significantly higher than those in healthy controls and patients with chronic hepatitis B (CHB) (Hu et al., 2017). Hepatocyte-derived HMGB1 is also involved in liver fibrosis. Blocking HMGB1 can partially prevent the consequences of mouse CCL4-induced liver fibrosis (Zhang et al., 2018). Moreover, the experiment targeting HMGB1 demonstrated it was a good therapeutic target for liver failure (LF) (Yamamoto and Tajima, 2017).

HMGB1 release induced by hepatic ischemic injury involves TLR-4-dependent reactive oxygen species (ROS) production and calcium-mediated signaling (Zhang et al., 2014). Due to the predominant role of hepatocytes in the biotransformation and metabolism of xenobiotics, ROS production constitutes a severe burden in liver pathophysiology in the progression of liver diseases (Klotz and Steinbrenner, 2017). The oxidized HMGB1 mediates apoptosis, and the production of HMGB1 is also a common downstream factor for multiorgan damage caused by apoptosis (Bai et al., 2017; Petrovic et al., 2017).

**Figure 1:**
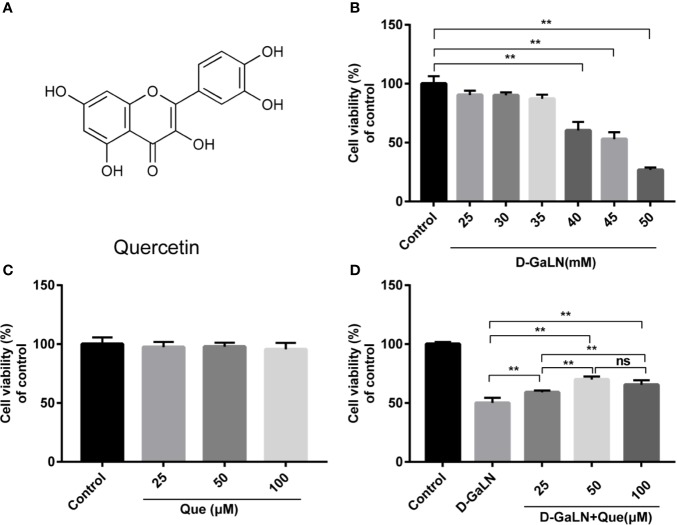
Protective effect of quercetin (Que) on d-galactosamine (d-GaLN)-induced cytotoxicity in L02 cells. **(A)** The chemical structure of Que. **(B)** Cells were treated with different concentrations of d-GaLN (25, 30, 35, 40, 45, 50 mM) **(C)** or Que (25, 50, 100 μM) for 12 h. **(D)** Cells were pre-treated with Que (25, 50, 100 μM) for 12 h and then co-treated with d-GaLN (45 mM) for 12 h. A Cell Counting Kit-8 (CCK8) assay was used to analyze cell viability. Data are presented as the mean ± SD,(*p < 0.05, **p < 0.01, n = 6);”ns” indicates not significant (*p* > 0.05).

Quercetin (Que) (3,5,7,3′,4′-pentahydroxyflavone) (**Figure 1**) is a typical flavonol-type flavonoid commonly found in vegetables, fruits, nuts, beverages, and traditional Chinese herbs (Darband et al., 2018). Que has been reported to possess a broad array of biological effects, including antioxidative, anti-inflammatory, and anti-apoptotic effects (de Oliveira et al., 2016; Zheng et al., 2017). It is now largely utilized as a nutritional supplement and as a phytochemical remedy for a variety of hepatic diseases like hepatitis, cirrhosis, acute liver failure, alcoholic or non-alcoholic fatty liver disease, and fibrosis (Miltonprabu et al., 2017; Li et al., 2018). Que has exhibited strong defensive effects against apoptosis, inflammation, and ROS generation in the liver of experimental animals exposed to various hepatotoxicants (Zou et al., 2015; Wang et al., 2017).

As an antioxidant, Que is also considered to be an inhibitor of HMGB1 (Li et al., 2016). However, it is not well known if the hepatoprotective effect of Que occurs through the antagonism of HMGB1 and the ensuing molecular signaling events. Therefore, the aim of this study was to investigate whether Que could protect L02 cells by inhibiting HMGB1, in addition to examining the underlying mechanism of Que, in order to provide a theoretical basis for Que as a hepatoprotective drug targeting HMGB1.

## Materials and Methods

### Chemicals and Reagents

Quercetin was obtained from Sigma-Aldrich (St. Louis, USA; cat: Q4951); its purity ≥ 95%. d-Galactosamine (**d**-GaLN; cat: G1639) and dimethyl sulfoxide (DMSO; cat: D2650) were also obtained from Sigma-Aldrich (St. Louis, USA). Anti-HMGB1 (cat: ab79823), anti-TLR-4 (cat: ab13867), anti-NF-κB p65 (cat: ab32536), anti-iNOS (cat: ab178945), anti-COX-2 (cat: ab179800), anti-Bcl-2 (cat: ab182858), anti-caspase-9 (cat: ab202068), and anti-caspase-3 (cat: ab184787) antibodies were obtained from Abcam (Shanghai, China).

### Cell Culture and Treatment

Normal human hepatocytes (L02 cells) obtained from the Cell Bank of Type Culture Collection of the Chinese Academy of Sciences (Shanghai, China) were maintained in DMEM media (HyClone, USA; cat: SH30243.01) supplemented with 10% (v/v) fetal bovine serum (FBS) (ExCell Bio, China; cat: FSP500), streptomycin at 37°C in a humidified atmosphere with 5% CO_2_. A Que stock solution was prepared in DMSO and diluted with culture media immediately prior to the experiment. Control cells were treated with an equal amount of DMSO alone at a final concentration of <0.1% (v/v).

### Cell Viability Assay

To evaluate the IC50 of d-GaLN and the noncytotoxic concentration of Que on L02 cells, the effects of d-GaLN and Que on the viability of L02 cells were evaluated and counted using a Cell Counting Kit-8 (CCK-8) assay (Dojindo Laboratories, Japan; cat: CK04), according to the manufacturer’s instructions. Briefly, cells were grown on 96-well plates at a density of 1 × 104 for 12 h. After treatment with Que and/or d-GaLN for the indicated time, the cells were incubated with 10 μl of the CCK-8 solution. After incubation at 37°C for 2 h in a humidified CO2 incubator, the absorbance was monitored at 450 nm on a microplate reader (Thermo Scientific, USA). The cell viability was calculated by comparing the optical densities of samples to the control (media only) cells. The optical density of the formazan formed in control cells was taken as 100% viability.

### TUNEL

The apoptotic response of L02 cells was identified using a TUNEL assay and a Fluorescein In Situ Cell Death Assay Kit (KeyGEN BioTECH, China; cat: KGA7072) according to the manufacturer’s instructions. The cells were cultured in a 12-well plate; after exposure to the desired experimental conditions, and L02 cells were fixed with 4% paraformaldehyde (PFA) for 30 min. After washing with PBS, 0.1% Triton X-100 was allowed to permeate for 5 min. With further washing, the reaction was carried out in a terminal deoxynucleotidyl transferase (TdT) buffer with fluorescein-labeled dUTP. The sample was then incubated with reagents at 37°C for 1 h in the dark with sealing to avoid evaporation of the reagents. After washing with PBS, the coverslips were mounted with an anti-fluorescent quenching sealer containing DAPI. The images were observed with a confocal laser scanning biomicroscope (Leica TCS SP8).

### Flow Cytometry Analysis of Apoptosis

The ratios of apoptotic cells were measured with an Annexin V-FITC/PI Apoptosis Detection Kit (KeyGEN BioTECH, China; cat: KGA108). Briefly, after exposure to the desired experimental conditions, L02 cells were collected by trypsinization and centrifugation. Then, the cells were resuspended at room temperature and fixed in a solution of a binding buffer (195 μl of annexin V-FITC, 5 μl of annexin FITC, and 10 μl of propidium iodide (PI)) for 15 min in the dark. The percentages of apoptotic cells were analyzed by flow cytometry (BD LSR Fortessa). The apoptotic rate is the apoptotic cells/all cells.

### Measurement of ROS

Intracellular ROS production was measured using an ROS assay kit (Beyotime, China; cat: S0033). The L02 cells were exposed to the desired experimental conditions, and the positive control group was incubated with Rosup for 30 min. The cells were then incubated with 10 μM CFH-DA for 30 min at 37°C. Then, the cells were collected and washed with serum-free DMEM, and ROS levels were measured by flow cytometry (BD LSR Fortessa).

### Western Blot

Whole cell lysis was obtained using a RIPA lysis buffer and protease inhibitor according to the user’s protocol. Cytoplasmic and nuclear proteins were isolated using nuclear and cytoplasmic protein extraction kits (Beyotime, China; cat: P0028), according to the manufacturer’s instructions. Protein concentration was determined using a BCA protein assay kit. An equal amount of protein (30 μg) was separated by 10% to 15% SDS-PAGE and then electrotransferred onto a PVDF membrane. The membrane was blocked with 5% skim milk for 1 h and then incubated overnight at 4°C with the following antibodies: HMGB1, TLR4, caspase-3, caspase-9, Bax, Bcl-2, NF-κB, p65, iNOS, and COX-2. Then, the membrane was incubated with a secondary antibody for 1 h at room temperature. After washing 3 times with TBST, the reaction was detected with an enhanced chemiluminescent reagent (NCM Biotech, China; cat: P10100). A ImageQuantLAS4000 Chemiluminescence Imaging system was used to visualize the target proteins (GE Co., USA) and densitometry was performed using the Image J software version 1.80. Some results of western blot were presented in three technical replicates, the repeatability has been confirmed by independent experiments.

### Immunofluorescence

Cells were seeded in 12-well plates and after exposure to the desired experimental conditions, were fixed in 4% PFA for 30 min, permeabilized in 0.5% Triton X-100 buffer for 20 min, and blocked with 5% BSA for 30 min. They were then incubated with a primary antibody (rabbit anti-human HMGB1 and rabbit anti-human TLR-4) at 4°C overnight, with the secondary antibody (FITC/TRITC-conjugated goat anti-rabbit IgG) incubated for 1 h. After being washed with PBST, slides were covered with an anti-fluorescent quenching sealer containing DAPI. Images were observed with a confocal laser scanning biomicroscope (Leica TCS SP8).

### RNA Interference

Short hairpin RNA (shRNA) was applied to silence HMGB1 at the mRNA level, as well as at the protein level. The HMGB1 sh-RNA (5′-GCT CAAGGAGAATTTGTAA-3′) plasmid vectors (sh-HMGB1) were purchased from GeneCopoeia (Guangdong, China) and transfected into L02 cells with a negative control (sh-NC) using Lipofectamine™ 2000 (Invitrogen, Thermo Fisher Scientific, Inc.; cat: 11668019). After transfection for 36 h, qRT-PCR and immunoblotting assays were conducted to estimate the transfection efficiency.

### Real-Time PCR

To detect the expression of HMGB1 mRNA, total RNA was extracted from the stably transfected cells using a RNAprep Pure Cell/Bacteria Kit (Tiangen, Beijing, China; cat: DP430), and cDNA was synthesized using a reverse transcriptase kit (Tiangen, Beijing, China; cat: KR116-02). The HMGB1 forward and reverse primers were 5′-ATATGGCAAAAGCGGACAAG-3′ and 5′-GCAACATCACCAATGGACAG-3′. The β-actin forward and reverse primers were 5′-TGGCACCCA GCACAATGAA-3′ and 5′-CTAAGTCATAGTCCGCCTAG AAGCA-3′. The melting curve data were analyzed to determine PCR specificity. Relative fold expressions were analyzed using the 2−ΔΔCt method, using β-actin Ct values as the internal reference in each sample.

### Molecular Docking Simulations

Molecular docking method was used to study the binding mode of Que and HMGB1. The software Ledock with Lepro tools and the web server CB-Dock were used for performing molecular docking simulations (Zhang and Zhao, 2016; Liu et al., 2019) was obtained from the RCSB Protein Data Bank and a ligand file of Que in the MOL2 was obtained from the ZINC database. For LeDock, the receptor files were processed by the LePro tool. All parameters were set to default for sampling by a combination of simulated annealing and evolutionary optimization. Docking scores were calculated by the default scoring function. Ligplot software was used for 2D interaction visualization (Laskowski and Swindells, 2011). For CB-Dock, the two files were uploaded and submitted to the CB-Dock server. The result table listed Vina scores, cavity sizes, docking centers, and sizes of predicted cavities. Once a ligand in the table is selected, the structure in the interactive 3D graphics is visualized.

### Statistical Analysis

All experiments were repeated three times. The results were expressed as the mean ± standard deviation (SD). GraphPad 7.0 statistical software was utilized for the statistical analyses. *p* values were computed by ANOVA with Tukey’s *post hoc* test. *p* < 0.05 was regarded as statistically significant.

## Results

### Que Protects L02 Cells Against d-GaLN-Induced Injury

d-GaLN is a commonly used experimental drug for causing hepatotoxic damage (Dejager and Libert, 2008; Gehrke et al., 2018). The results (**Figure 1B**) indicate that d-GaLN significantly reduced the viability of L02 cells in a dose-dependent manner. Treatment with a concentration of 45 mM d-GaLN for 12 h lowered the cell viability to 52.92% ± 5.93%. Therefore, this concentration was used in subsequent experiments. To investigate the protective effects of Que, the results (**Figure 1C**) indicate that treatment with less than 100 μM Que did not result in significant cell death. Then, cells were pretreated with 25 to 100 μM of Que for 12 h and then with 45 mM d-GaLN. The results (**Figure 1D**) showed that 25, 50, and 100 μM of Que significantly attenuated d-GaLN-induced cell death. The protective effects of 50 and 100 μM were significantly higher than those of 25 μM. Although there was no statistical difference, the cell viability of 50 μM was higher than that of 100 μM. Therefore, it was determined that pretreatment with 50 μM Que for 12 h followed by incubation with 45 mM d-GaLN for 12 h was the optimal condition for the following experiments.

### Que Reduces L02 Cell Damage by Inhibiting ROS and Apoptosis

**Figure 2:**
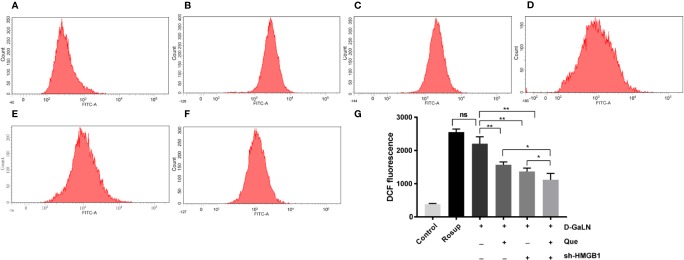
Flow cytometric analysis of the intracellular reactive oxygen species (ROS) levels in L02 cells. **(A)** Control group; **(B)** Rosup group; **(C)** Cells treated with d-GaLN (45 mM) alone; **(D)** Cells pre-treated with Que (50 μM) and then treated with d-GaLN (45 mM); **(E)** Cells pre-treated with sh-HMGB1 and then treated with d-GaLN (45 mM); **(F)** Cells pre-treated with sh-HMGB1 and Que (50 μM) and then treated with d-GaLN (45 mM); **(G)** Statistical results of ROS. Data are presented as the mean ± SD of three independent experiments (*p < 0.05, **p < 0.01); “ns” indicates not significant (*p* > 0.05).

We analyzed the ROS production in L02 cells by measuring the DCF fluorescence intensity. The results (**Figures 2A–C**) indicated that d-GaLN significantly increased intracellular ROS accumulation, while intracellular ROS in the Que group (**Figure 2D**) was significantly reduced compared to the d-GaLN group.

**Figure 3:**
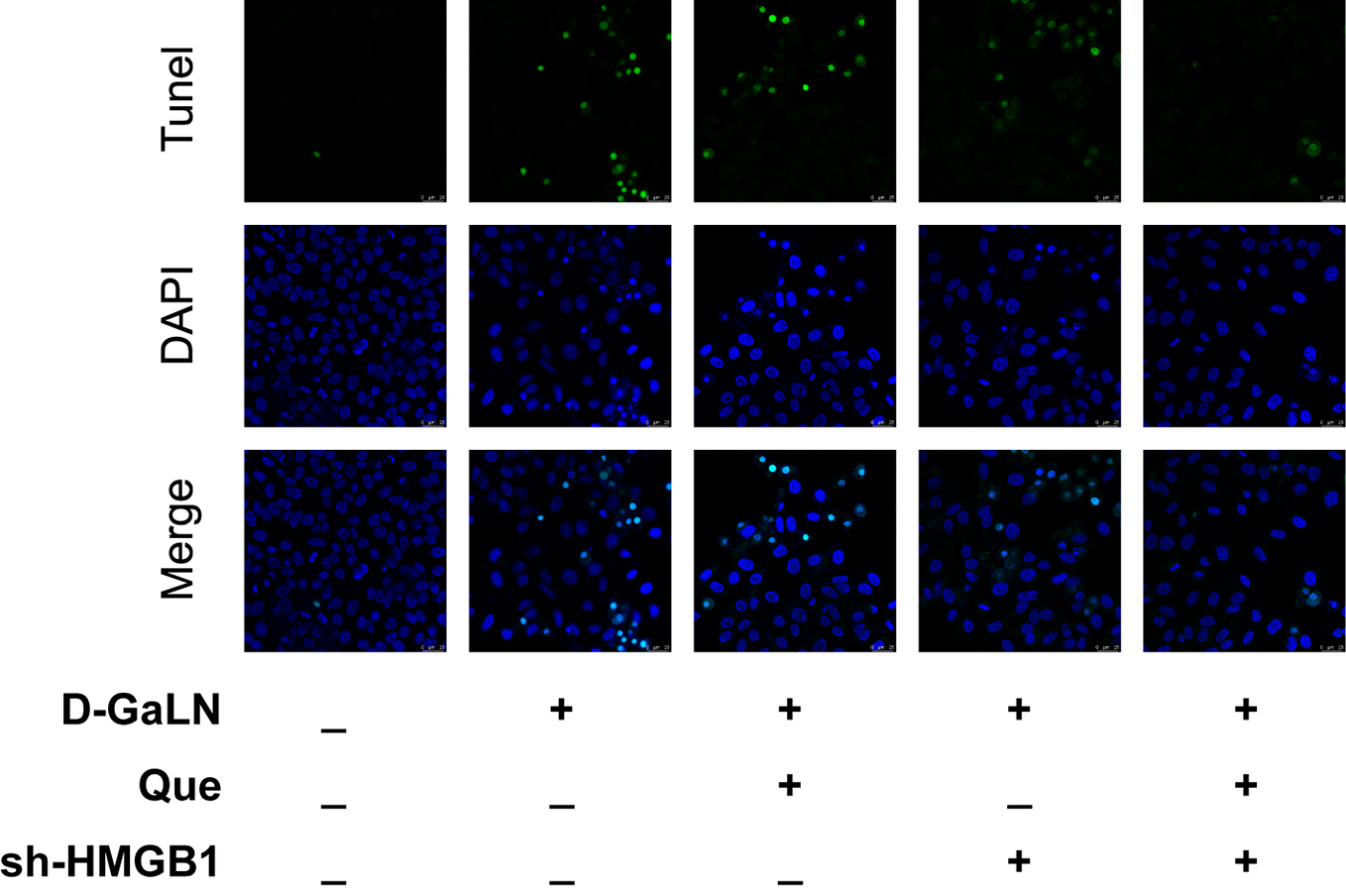
Cell apoptosis was identified by (TdT-mediated dUTP nick end labeling) TUNEL analysis in L02 cells. Green fluorescence represents TUNEL-positive cells. Scale bar: 25 μm. All nuclei were stained with 4′,6-diamidino-2-phenylindole (DAPI) (blue fluorescence).

**Figure 4:**
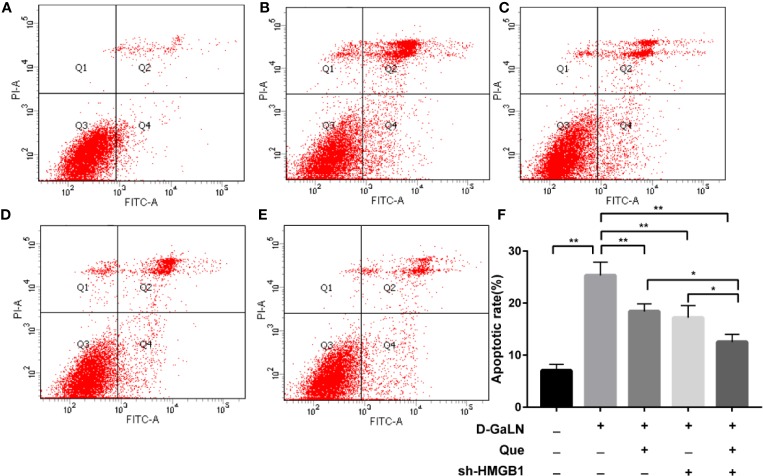
The apoptotic rate of L02 cells was evaluated by flow cytometry after staining with annexin V-FITC/PI. **(A)** Control group; **(B)** Cells treated with d-GaLN (45 mM) alone; **(C)** Cells pre-treated with Que (50 μM) and then treated with d-GaLN (45 mM); **(D)** Cells pre-treated with sh-HMGB1 and then treated with d-GaLN (45 mM); **(E)** Cells pre-treated with sh-HMGB1 and Que (50 μM) and then treated with d-GaLN (45 mM); **(F)** The results of the apoptotic rate. Data are presented as the mean ± SD of three independent experiments (*p < 0.05, **p < 0.01).

**Figure 5:**
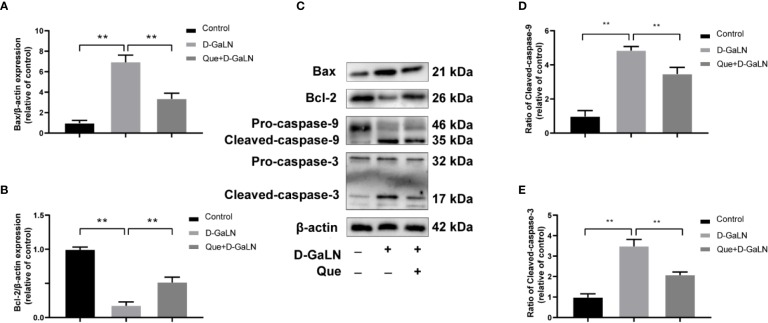
Effect of Que on the expression of d-GaLN-induced apoptosis-related proteins. **(A**, **B**, **D**, **E)** The relative expression levels of Bax/β-actin, Bcl-2/β-actin, ratio of cleaved caspase-9, and ratio of cleaved caspase-3. The data on quantified protein expressions were normalized by related β-actin (fold change of control); **(C)** Representative immunoblots for the Bax, Bcl-2, caspase-9, caspase-3, and β-actin proteins. Data are presented as the mean ± SD of three technical replicates (**p < 0.01).

Next, to determine the effect on apoptosis, we used a TdT-mediated dUTP nick end labeling (TUNEL) assay to observe the apoptosis caused by d-GaLN and calculate the apoptotic rates with annexin V-FITC/PI. d-GaLN significantly caused apoptosis in L02 cells (**Figure 3**) and increased apoptotic rates (**Figures 4A, B**). However, compared with the d-GaLN group, Que significantly reduced the increased apoptosis rate (**Figure 4C**). We further used Western blot to examine the effects of d-GaLN and Que on the expression of Bcl-2, Bax, caspase-9, and caspase-3 proteins in L02 cells (**Figure 5**). The results show that d-GaLN significantly increased the expression of Bax, ratio of cleaved caspase-9 and cleaved caspase-3, but decreased the expression of Bcl-2 in L02 cells. Compared with the d-GaLN group, Bax, ratio of cleaved caspase-9 and cleaved caspase-3 were decreased in the Que group, while Bcl-2 expression was increased. These results showed that Que can weaken d-GaLN-induced oxidative stress damage and apoptosis in L02 cells.

### HMGB1 Aggravates d-GaLN-Induced L02 Cell Damage

**Figure 6:**
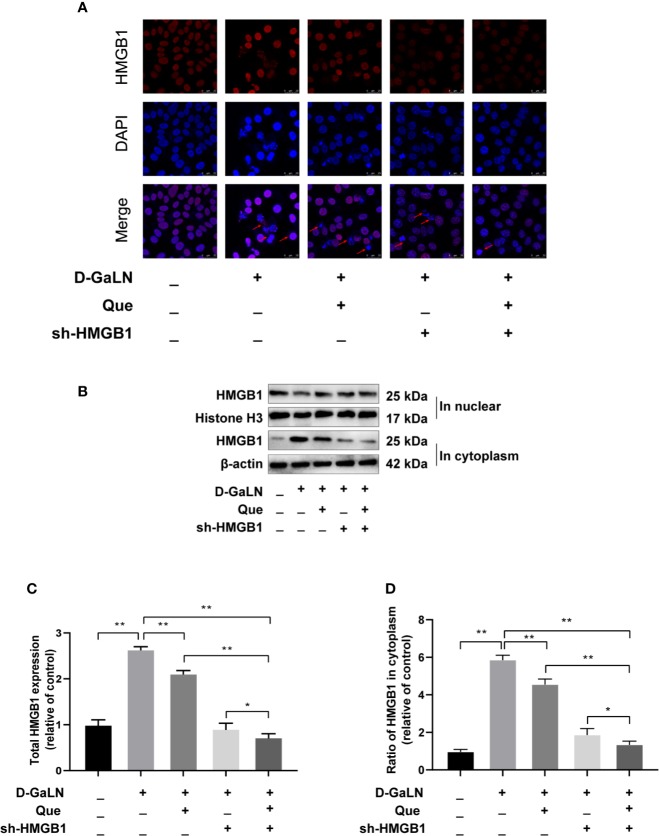
Effect of Que on d-GaLN-induced HMGB1 expression. **(A)** Immunofluorescence staining of HMGB1 expression under different treatment conditions. Scale bar: 25 μm; Arrows indicate the HMGB1. **(B)** Representative immunoblots for the HMGB1 in the nucleus, Histone H3, HMGB1 in the cytoplasm, and β-actin proteins. **(C**, **D)** Total HMGB1 and ratio of HMGB1 in cytoplasm under different exposure conditions by Western blot assay. The data on HMGB1 expression in the cytoplasm were normalized by related β-actin proteins, HMGB1 in the nucleus were normalized by related Histone H3 proteins (fold change of control). Data are presented as the mean ± SD of three technical replicates(*p < 0.05, **p < 0.01).

Previous studies have reported that HMGB1 aggravates damage to hepatocytes, while HMGB1 is also involved in apoptosis (Zhao et al., 2017; Wu et al., 2018). Thus, we investigated the effects of Que on HMGB1, also the expression of its receptor (TLR-4) by Western blot and immunofluorescence (IF). **Figure 6** showed the increased expression of HMGB1 due to d-GaLN stimulation. Western blot also confirmed that due to the stimulation of d-GaLN, the total amount of HMGB1 and the ratio of HMGB1 in cytoplasm of L02 cells were increased. Moreover, the expression of TLR-4 in the d-GaLN group was significantly increased (**Figures 8B, C**), IF showed that TLR-4 was abundantly expressed in the cytoplasm and accumulated around the nucleus (**Figure 8A**). These indicated that the expression of HMGB1 was increased due to the stimulation of d-GaLN, which also affected the expression and distribution of its TLR-4 receptor.

**Figure 7:**
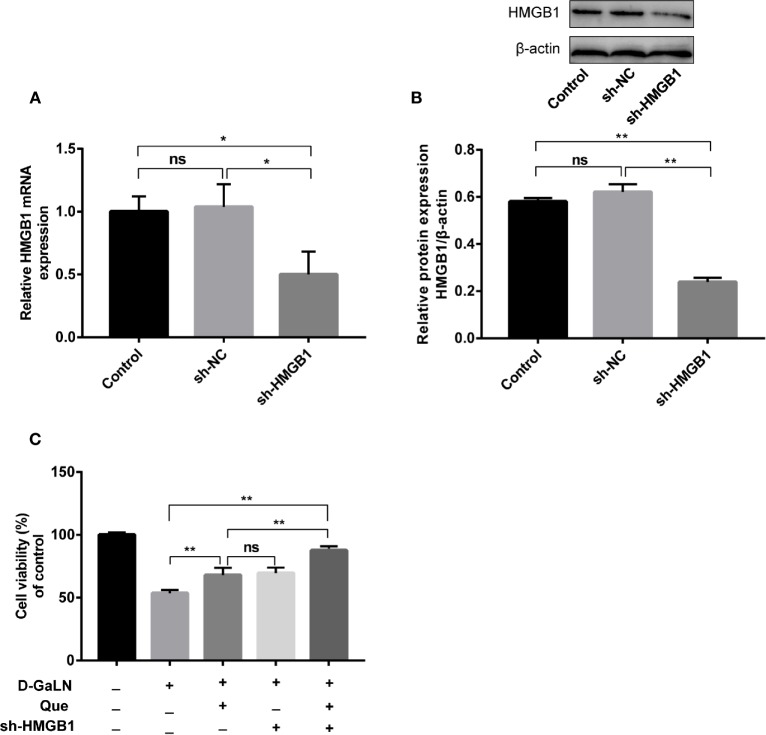
The effect of Que on the viability of L02 cells after silenced HMGB1. The sh-HMGB1 was transfected into L02 cells, and its transfection efficiency was confirmed at **(A)** the mRNA level, by qRT-PCR; **(B)** and the protein level, by Western blot assay; **(C)** after silenced HMGB1, the CCK8 assay was used to analyze the cell viability of L02 cells under different exposure conditions. Data are presented as the mean ± SD (*p < 0.05, **p < 0.01, n = 3); “ns” indicates not significant (*p* > 0.05).

**Figure 8:**
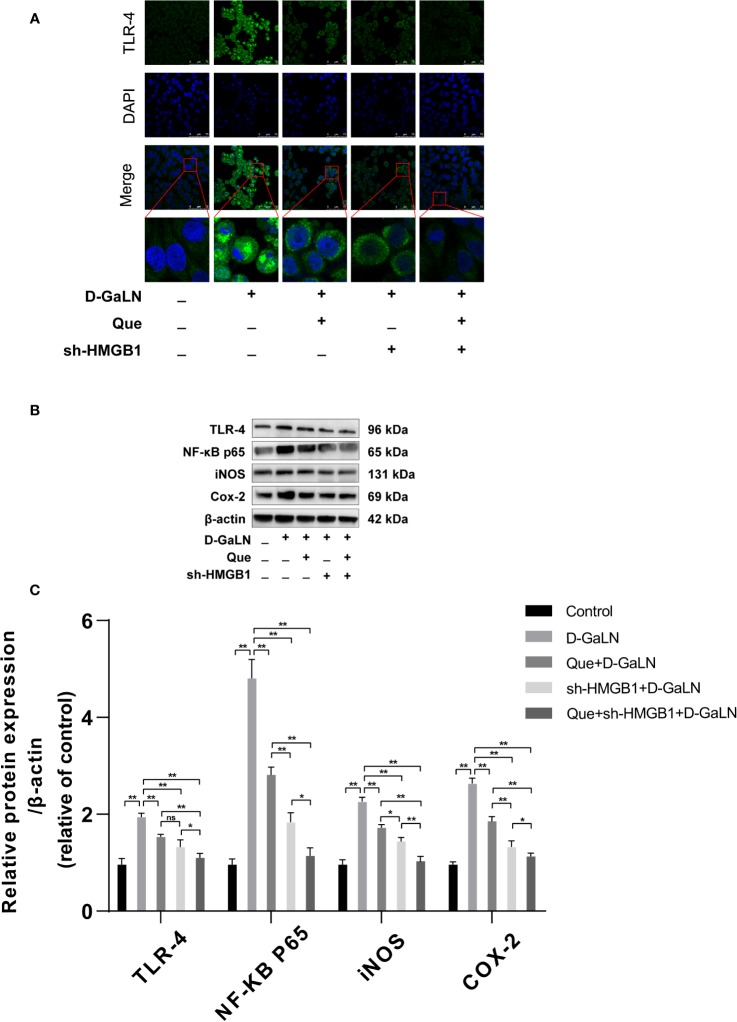
The effect of Que on the HMGB1 signaling pathway. **(A)** Immunofluorescence staining of TLR-4 receptor expression under different treatment conditions. Scale bar: 75 μm; **(B, C**) the TLR-4, NF-κB P65, iNOS, and COX-2 protein expression levels were evaluated by Western blot assay. The data on quantified protein expressions were normalized by related β-actin proteins. (fold change of control). Data are presented as the mean ± SD of three technical replicates (*p < 0.05, **p < 0.01).

Thus, we investigated the function of HMGB1 in cell injury by d-GaLN. Cells were transfected with short hairpin RNAs (shRNA) targeting HMGB1. The sh-HMGB1 could achieve a reduction of ~50% for HMGB1 expression (**Figures 7A, B**). As shown in **Figure 6**, sh-HMGB1 downregulated the expression of HMGB1. And the expression of TLR-4 receptors in L02 cell injury caused by d-GaLN was also decreased (**Figure 8**). The results of CCK-8 (**Figure 7C**), TUNEL (**Figure 3**), and flow cytometry (**Figures 2E** and **4D**) showed that sh-HMGB1 attenuated the loss of cell viability, inhibited intracellular ROS accumulation and apoptosis in d-GaLN-stimulated L02 cells. Consequently, HMGB1 seemed to be related to the injury caused by d-GaLN and may be involved in oxidative stress and apoptosis processes.

### Silencing of HMGB1 Enhances the Effect of Que on d-GaLN-induced L02 Cells

The results of the CCK8 assay (**Figure 7C**), TUNEL (**Figure 3**), and flow cytometry (**Figures 2F, G** and **4E, F**) confirmed that compared with Que and sh-HMGB1 alone, co-treatment with Que and sh-HMGB1, cell viability, ROS accumulation, and apoptosis were significantly ameliorated. We further examined changes in the expression of HMGB1 (**Figure 6**), when co-treated with Que and sh-HMGB1, the total amount of HMGB1 and the ratio of HMGB1 in the cytoplasm of L02 cells were decreased significantly. After silenced HMGB1, we analyzed changes in protein expression of related pathways by Western blot. Sh-HMGB1 significantly downregulated the expression of TLR-4, NF-κB-p65, iNOS, and Cox-2, similar to Que (**Figure 8**). Compared with Que and d-GaLN alone, when co-treated with Que and sh-HMGB1, the inhibition of TLR-4 receptor expression and other pathway proteins were significantly enhanced (**Figure 8**). These results indicated that Que attenuated d-GaLN-induced L02 cell damage by suppressing ROS generation and apoptosis, which may be controlled by the inhibition of HMGB1.

### Structural Details of the Interaction Between HMGB1 and Que

**Figure 9:**
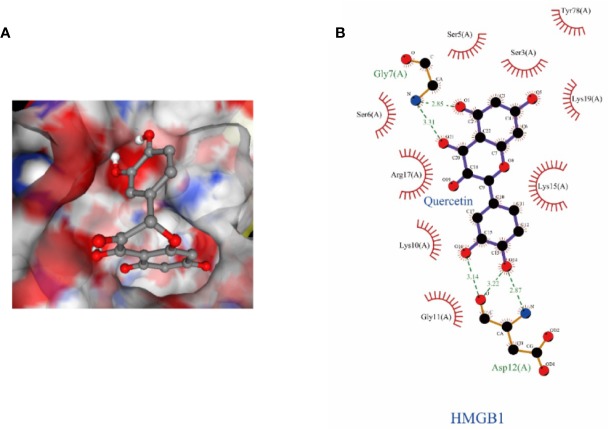
The structural details of the interaction between HMGB1 and Que were obtained by the docking method. **(A)** Surface representation of HMGB1-Que complex. **(B)** The interaction zone with Que showed the residues of interactions.

To better understand the molecular mechanism of Que on HMGB1, molecular docking studies on Que were performed. Molecular docking assays showed that Que can bind to the active pocket of HMGB1 crystal structure (**Figure 9A**) and form two stable hydrogen bonds with the Gly7 and Asp12 amino residues of HMGB1 (**Figure 9B**). Logarithms of free binding energy calculated by Ledock and CB-Dock were −6.23 and −7.1 kcal/mol. The interaction between Que and HMGB1 may affect the conformation of HMGB1, thereby inducing its downstream signal transduction.

## Discussion

d-GaLN is a commonly used experimental drug that causes hepatotoxicity, and its mechanism involves GSH depletion and affecting RNA synthesis (Gehrke et al., 2018). Studies have also confirmed that d-GaLN alone leads to the potent intracellular generation of ROS in HepG2 cells (Bak et al., 2018) which can induce oxidative stress in the accumulation of ROS in hepatocytes *in vitro* and *in vivo* (Gonzalez et al., 2009; Wen et al., 2018). By inducing caspase-3 activation and DNA fragmentation in hepatocytes, d-GalN causes hepatocyte apoptosis (Lin et al., 2009). Mitochondria are the source and target of ROS (Zorov et al., 2014), and excessive production of ROS leads to the apoptosis of hepatocytes (Hong et al., 2018), and d-GaLN causes apoptosis in a manner closely related to this pathway. The results of this experiment showed that d-GaLN affected the viability of L02 cells, causing ROS accumulation and mitochondrial apoptosis.

HMGB1 acts as a DAMP factor, playing an important role in various liver injuries (Geng et al., 2015). It has a significantly greater increase than chronic hepatitis, particularly in severe liver injury (Zhou et al., 2011). However, previous studies have mostly focused on the role of HMGB1 as a pro-inflammatory factor (Wang et al., 1999), but in addition to inflammation, excessive apoptosis is also an important mechanism of cell death in liver failure (Komarov et al., 2016). Blocking HMGB1 can inhibit caspase-3 activation, thereby reducing apoptosis (Tan et al., 2018). Recent studies have shown that apoptotic cells activate macrophages to release HMGB1 (Velegraki et al., 2013). HMGB1 interacts with phosphatidylserine on the surface of apoptotic neutrophils, thereby inhibiting the clearance of neutrophils by macrophage phagocytic cells (Liu et al., 2008). By binding to DNA, late-stage apoptotic cells can release HMGB1 (Shi et al., 2018); the release of HMGB1 was also present in cells with late apoptosis (Pisetsky, 2014) After being released, caspase-3 can be activated by the HMGB1–TLR4 pathway, resulting in apoptosis (Zhang et al., 2019).

Meanwhile, there is also evidence to confirm that HMGB1 is essential for oxidative stress (Tang et al., 2011). In vitro, recombinant HMGB1 leads to the TLR-4-dependent activation of NADPH oxidase and increased ROS production (Zhang et al., 2015). TLR-4-dependent ROS production and calcium-mediated signaling are involved in the HMGB1 release induced by liver ischemia (Tsung et al., 2007). Further, the mitochondria play an important role in apoptosis by relocating intermembrane mitochondrial proteins, such as Bcl-2 and Bax (Bhola and Letai, 2016). The induction of ROS can regulate mitochondrial membrane potential, leading to apoptosis initiation in the mitochondrial pathway (Sinha et al., 2013). Therefore, we hypothesize that HMGB1-mediated apoptosis is caused by the mitochondrial release of apoptotic proteins caused by ROS. To the best of our knowledge, the results of this experiment have demonstrated, for the first time, that HMGB1 participated in d-GaLN-induced L02 cell injury. In the injured L02 cells, the expression of its TLR-4 receptor and signaling pathway factors also increased accordingly. Moreover, after silenced HMGB1, ROS production and apoptosis were significantly improved. Therefore, HMGB1 is closely related to the occurrence of oxidative stress and mitochondrial apoptosis.

Que is a commonly used dietary supplement flavonoid (D’Andrea, 2015); it can alleviate acute liver injury induced by lipopolysaccharide (LPS)/d-GalN through anti-inflammatory, antioxidative, and anti-apoptotic activity (He et al., 2019). Que also prevents oleic acid-induced ROS production and mitochondrial damage in HepG2 hepatocytes (Rafiei et al., 2019) and can protect against oxidative stress by inhibiting the iNOS/NF-κB pathway (Bahar et al., 2017). Meanwhile, Que is also a potential inhibitor of HMGB1 (Musumeci et al., 2014). In this experiment, we demonstrated that Que protects L02 cells from damage caused by d-GaLN. Moreover, we confirmed for the first time that the protective effect of Que on L02 cells is produced by inhibiting HMGB1 and subsequent oxidative stress and mitochondrial apoptosis mediated by the associated signaling pathway. And molecular docking showed that the high affinity of Que and HMGB1 is related to the hydrogen bonding with Gly7 and Asp12 residues. This binding may affect the conformation of HMGB1, thereby inducing its downstream signal transduction.

## Conclusion

In conclusion, our current results indicate that HMGB1 was involved in d-GaLN-induced L02 cell injury. However, Que can inhibit HMGB1 to protect L02 cells from d-GaLN-mediated damage. This protective effect is associated with the inhibition of oxidative stress and mitochondrial apoptosis mediated by HMGB1. Further, molecular docking showed that hydrogen bonding with Gly7 and Asp12 residues are involved in the binding of Que and HMGB1. Therefore, our experimental results provide a theoretical basis for using Que as a hepatoprotective drug targeting HMGB1.

## Data Availability Statement

All datasets generated for this study are included in the article/supplementary material.

## Author Contributions

PF and QZ conceived and designed the experiments. JL, XW, XJ, XF, MW, XW, WY, and WH were involved in the experimental study design, preparation, and review of this manuscript. All authors have reviewed and approved the final version of the manuscript.

## Funding

This research was funded by the National Natural Science Foundation of China (Grant no. 81573767), Natural Science Foundation of Beijing Municipality (Grant no. 7192024).

## Conflict of Interest

The authors declare that the research was conducted in the absence of any commercial or financial relationships that could be construed as a potential conflict of interest.
